# A Robust Dirichlet Reputation and Trust Evaluation of Nodes in Mobile Ad Hoc Networks

**DOI:** 10.3390/s22020571

**Published:** 2022-01-12

**Authors:** Eric Chiejina, Hannan Xiao, Bruce Christianson, Alexios Mylonas, Chidinma Chiejina

**Affiliations:** School of Physics, Engineering, and Computer Science, University of Hertfordshire, College Lane, Hatfield AL10 9AB, Hertfordshire, UK; h.xiao@herts.ac.uk (H.X.); b.christianson@herts.ac.uk (B.C.); a.mylonas@herts.ac.uk (A.M.); c.o.chiejina2@herts.ac.uk (C.C.)

**Keywords:** cooperative nodes, reputation, trust, candour, total reputation, trust values, trustworthiness, MANETs

## Abstract

The distributed nature of mobile ad hoc networks (MANETs) presents security challenges and vulnerabilities which sometimes lead to several forms of attacks. To improve the security in MANETs, reputation and trust management systems (RTMS) have been developed to mitigate some attacks and threats arising from abnormal behaviours of nodes in networks. Generally, most reputation and trust systems in MANETs focus mainly on penalising uncooperative network nodes. It is a known fact that nodes in MANETs have limited energy resources and as such, the continuous collaboration of cooperative nodes will lead to energy exhaustion. This paper develops and evaluates a robust Dirichlet reputation and trust management system which measures and models the reputation and trust of nodes in the network, and it incorporates candour into the mode of operations of the RTMS without undermining network security. The proposed RTMS employs Dirichlet probability distribution in modelling the individual reputation of nodes and the trust of each node is computed based on the node’s actual network performance and the accuracy of the second-hand reputations it gives about other nodes. The paper also presents a novel candour two-dimensional trustworthiness evaluation technique that categorises the behaviours of nodes based on their evaluated total reputation and trust values. The evaluation and analyses of some of the simulated behaviours of nodes in the deployed MANETs show that the candour two-dimensional trustworthiness evaluation technique is an effective technique that encourages and caters to nodes that continuously contribute to the network despite the reduction in their energy levels.

## 1. Introduction

The absence of centralised authority in a Mobile Ad Hoc Network (MANETs), poses a key challenge due to the dire need for cooperative network operation amongst nodes. In MANETs, some nodes may exhibit behaviours that negate the routing protocol functionality through the disruption of the route discovery process [1]. To ensure that data is readily available in a MANET, all nodes must function as a forwarder and participate in the transmission of data packets from the source to the desired destinations [2]. MANETs can generally be set up anywhere and anytime due to their dynamic nature. However, as a result of their unique characteristics, MANETs are more vulnerable to various security threats [3] such as grey-hole attacks, black-hole attacks, eavesdropping, denial of service attacks (DoS), etc. when compared to traditional networks.

Security in MANETs generally involves ensuring and maintaining the integrity and confidentiality of data, in addition to the legitimate use and availability of network service provided by each network node [1]. The viability of a MANET is solely dependent on the reliability of nodes to actively participate in the route discovery processes and to honestly forward data packets for other nodes in the network. To attain optimal network performance, each node must continuously forward packets for nodes within its radio range when required. Forwarding or routing of data packets by a node requires the consumption of its limited energy without expecting any rewards for its actions. A situation where a significant number of nodes in a MANET selfishly decides to preserve their energy level by minimizing their network participation such as not actively responding to route requests or not forwarding data packets [2], could lead to a degraded network performance [3], and one of the main goals of designing and creating a MANET, i.e., to support vigorous and effective routing operations by ordinary nodes is defeated. Thus, there is a dire need for an efficient reputation and trust management system (RTMS) which encourages the active collaboration of nodes in the network. A lot of existing works on Reputation and Trust Management systems in MANETs [4] enforced the collaboration of nodes by isolating and repudiating uncooperative nodes from the available network resources. These RTM systems focused primarily on modelling effective mechanisms that ensure collaboration among nodes and they usually consider no punitive measure as a form of incentive for the cooperative nodes in the network [5,6,7,8,9].

In general, the cooperative nodes in most of the reviewed RTM systems do not reward nodes for the continuous utilization of their limited energy in forwarding packets for other nodes. Nodes that actively participate in route discovery processes and the forwarding of data packets tends to experience low energy levels after a certain period. These low energy levels may in turn hamper their ability to carry out successful network operations which in turn may have a negative effect on their respective reputation and trust as well as their network performance [4].

These cooperative nodes usually end up getting penalised by the mechanisms deployed by these RTMs models for not being able to continuously carry out the expected network activities. This process of punishing active nodes after a long period of contributing to successful network operations is unfair. It is a known fact that nodes do not have unlimited energy levels and thus, there is a dire need for a reliable reputation and trust management system that would enforce cooperation by ensuring that collaborative nodes are rewarded for continuously conducting favourable network operations while nodes that are judged to be selfish or malicious nodes are penalised, isolated or denied the available the network resources. This concept of rewarding nodes for continuously carrying out favourable network operations was initially proposed by Chiejina et al. [4]. The authors suggested a conceptual RTM model in which nodes that are judged to be trustworthy will be rewarded for their active network participation while untrustworthy nodes will be penalised in the network using a two-dimensional approach. However, this concept was not fully explored in their paper.

In this paper, we adopted the initial concept proposed in [4] and we present a candour two-dimensional trustworthiness evaluation technique to determine the trustworthiness of nodes in MANETs. Our proposed RTM models the reputation and trust evaluation of nodes by ensuring both positive and negative behaviours exhibited by a node are considered before the trustworthiness of the node can be determined. This paper also explores the following:

- Possible ways of understanding nodes’ behaviours in a MANET without bias
- How the use of observed optimal weights of a node at any given time will enable candour in the trustworthiness evaluation of nodes in the proposed RTM model
- How candour, which is the ability to make unbiased trust-aware decisions can be incorporated into reputation and trust management systems in MANETs.

In an overview, the proposed RTM system models the first-hand reputation of a target node using Dirichlet probability distribution. This idea was based on the discovery that the Dirichlet probability distribution provides a platform for designing a practical reputation system that enables the behaviours of nodes to be expressed using more than two possibilities. This allowed the observed behaviours of nodes in our proposed model to be measured from three possible natures which were termed benevolence, selfishness, and maliciousness. Furthermore, the novel candour two-dimensional trustworthiness evaluation technique presented in this paper is based on what a node says about other nodes and what it does with regard to forwarding packets. The observed optimal weights at any given time were recommended to be used in evaluating the trustworthiness of a node which would ensure that nodes are not unfairly penalized especially if they can still contribute to the network passively (by providing genuine second-hand reputations about other nodes).

The rest of the paper is organised as follows: Section 2 contains related works on reputation and trust management systems in MANETs. Section 3 discusses a two-dimensional view of a node’s network activities where node behaviours and the node categorization. Section 4 and Section 5 presents the proposed robust Dirichlet reputation and trust evaluation of Nodes in Mobile Ad Hoc Networks. Section 6 presents details of the implementation work. Section 7 presents the simulation results, and the analysis. Section 8 presents the discussions and further analyses, and Section 9 concludes by setting out the benefits of the proposed system and outlines future research works.

## 2. Related Works

Collaboration implementation in MANETs using the concept of reputation management systems has received considerable attention by researchers in the ad hoc network community over the past two decades of which a lot of research works have been proposed and carried out on reputation and trust and management (RTM). Most RTM models employ different monitoring techniques in gathering data, which are used in computing the reputation and trust of nodes in the networks [5,7,8,9,10,11,12,13,14,15,16,17,18,19,20,21,22,23,24,25,26,27,28,29]. Several publications have proposed various reputation management-based techniques in which nodes in MANETs monitor the packet forwarding activities of their neighbours. If a node contributes towards forwarding packets for other nodes, the reputation of the node is computed and increased [5,7,8,9,10,11,12,13,14,15,16,17,18,19,20,21,22,23,24,25,26,27,28,29]. Similarly, if the nodes are observed dropping packets that are presented to them for forwarding, the RTM models decrease the reputation of such nodes. A significant number of these RTM models employ weight-based or threshold-based techniques in analysing the computed reputation values of the observed nodes in their respective networks before deciding on the observed nodes. In some cases, if a node’s reputation drops below a specified threshold or weight, the node is penalised which could include being isolated from the network or deprived of the available network resources as in the proposed RTM models in [5,17,19,28,29].

Li Yang et.al [30] proposed a Dirichlet reputation system for reliable routing of wireless ad hoc networks. In their proposed work, they employed the use of the Dirichlet reputation model which is solely based on Bayesian inference theory to model and evaluate the reliability of nodes in their network in terms of packet delivery. Their proposed model uses a unique mechanism to determine, predict and select a reliable routing path through a blend of first-hand observation and second-hand reputation reports. Simulation results show that their proposed reputation system could decrease the damaging effects triggered by misbehaving nodes and in turn improve the throughput of the network.

Sun et al. [31] proposed, designed, and implemented a trust model which is very effective in computing the trust level of observed nodes in the network using a probabilistic algorithm based on the uncertainty that a node being observed directly by its neighbours will carry out a specific action successfully, considering only the monitored information. Their proposed model was able to ensure that the routing of packets in the network is extra secure and it also improves the intrusion detection systems in the network.

In their proposed model, Na et al. [26] employed a trust-based architecture that includes a reputation system and a Watchdog. Their proposed model uses a Positive Feedback Message (PFM) as evidence of the forwarding behavior of a node, which is fed into the Watchdog. The watchdogs deployed in their models normally monitor the events of data forwarding and count the arrival of the acknowledgment packets (ACKs) with respect to the forwarded data. This mechanism is used to determine a node’s forwarding ability which translates to its defined trustworthiness.

In their proposed reputation model, Chiejina et al. [17] employs a novel direct monitoring technique to evaluate the reputation of a node in the network. Their model ensures that nodes that expend their energy in transmitting data and routing control packets for others can carry out their network activities while the misbehaving nodes are detected and isolated from the network. Simulation results show that their model is effective at curbing and mitigating the effects of misbehaving nodes in the network.

Additionally, Michiardi and Molva [24] proposed a collaborative reputation system known as the CORE. Their model consists of a watchdog component, which is enhanced with a reputation system that distinguishes between observations (subjective reputation), positives report by others (indirect reputation), and task-specific behaviours (functional reputation). These various reputations are then weighted to generate an aggregated trust value which is employed in making decisions about collaborating with trustworthy nodes or to slowly isolate malicious and misbehaving nodes from the network. The reputation values in their model are acquired by viewing all nodes as both requesters and providers of nodes’ behavioural activities and analysing the derived results from the expected results for each request. Nodes exchange periodic updates of only good reputation data. As a result, there is a compromise between robustness against false reports and the swiftness of detection. Since only positive reports are exchanged in the proposed model, a false-positive report will make it extremely difficult to detect and isolate malicious nodes in the network.

In their proposed model, Cho et al. [32] evaluated a trust management protocol for cognitive mission-driven Group communication systems in MANETs for expeditious development of satisfactory trust relationships between nodes that don’t have past interaction history among themselves. The authors outlined a composite trust algorithm which is a combination of social and Quality of Service (QoS) trust. This was achieved by applying a ranked Stochastic Petri Nets (SPN) model to depict the behaviour of a node with integrated intelligence to trade of trust space for trust level over a given period. Through numeric analysis, the authors were able to determine the best trust chain length to optimise the trust level of collaboration nodes on a given trust chain. Their model incorporated the unique characteristics of MANETs, and they were able to show that an utmost reliance on subjective reputation in computing trust will make a node more susceptible to risk and an utmost reliance on recommendations from other network nodes will allow conventional trust relationships which may lead to loss of cooperative opportunities.

In their proposed model, Buchegger and Boudec [33] analysed a RTM system for MANETs and peer-to-peer networks in which the authors used both direct and second-hand reports in computing reputation and trust values for nodes in a network. The authors critically analysed the effects of spreading rumour in a MANET and they were able to filter false reports from liars among nodes before calculating the respective reputation and trust values of the nodes. By using accurate second-hand reports, the authors were able to increase the robustness of their RTM system and speed up the detection rate of malicious nodes.

In their proposed model, He et al. [27] employed a secure and objective reputation-based incentive scheme for MANETs. The reputation of nodes in their proposed model is computed and quantified by objective measures, and the dissemination of reputation is carried out efficiently by a secured one-way-hash-chain-based authentication. Their proposed model also uses punitive measures as a way of encouraging packet forwarding and penalizing selfish nodes by probabilistically dropping packets that originate from those nodes.

After critically analysing and reviewing some of the above-mentioned related works to our proposed model, we identified some unaddressed issues in existing RTM systems [5,7,8,9,10,11,12,13,14,15,16,17,18,19,20,21,22,23,24,25,26,27,28,29] relating to fairness in its mode of operation of these RTM models. Our proposal considers that nodes have limited energy. Its functions cater to situations that may hamper an active node’s performance level due to low energy. It considers that genuine nodes in the network which are unable to actively forward packets due to low energy may still provide accurate recommendations. These recommendations usually require a low amount of energy to execute. Furthermore, the qualitative and quantitative node categorisation in existing RTM models has not been exhaustively analysed. Some past works on RTM systems [5,6,8,9,10,11,12,13,14,15,16,17,18,30] categorised nodes based on the good or bad behaviours they exhibited in the network. In this paper, we presented a categorisation in which a distinctive baseline is drawn between a node’s active network operations such as successful, or unsuccessful forwarding of packets, and its passive operations, such as the accuracy of the recommendations disseminated about other nodes. This research area has not been extensively investigated in past research work.

In addition, our proposed model used the observable optimal weights in evaluating the trustworthiness of a node in the network, to introduce and maintain candour in the trustworthy evaluation process of nodes from the computed total reputation and trust values, which is required in determining the true nature of a node from the computed values.

## 3. Two-Dimensional View of a Node’s Network Activities

**Figure 1:**
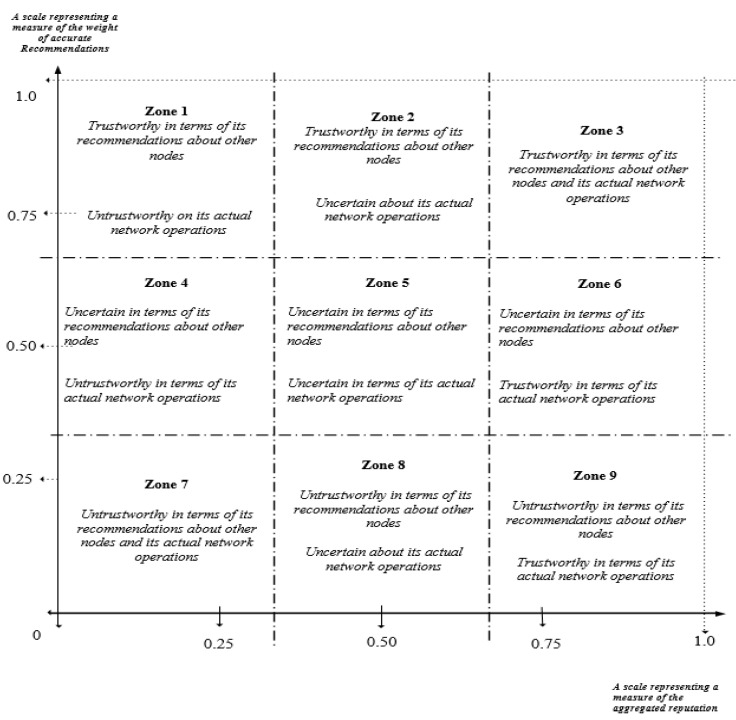
Two-dimensional view of trustworthiness evaluation of a node.

The two-dimensional view takes into account the node’s active network operations (i.e., forwarding packets for other nodes) which are used in evaluating the reputation of the node, and the passive activities such as sending 2nd-hand reputation to other nodes. A two-dimensional categorisation of nodes in which a node’s active network operations and the accuracy of its second-hand reputations about other nodes are used in evaluating the trustworthiness of a node. In Figure 1, the “*Y*-axis” represents the weight of the accuracy of second-hand reputations a node makes about other nodes and the “*X*-axis” represents the weight of the evaluated total reputation values of a node in the network. A node that falls into zones 1, 2, and 3 can be classified as being trustworthy because its second-hand reputations about other nodes are of high quality and very accurate. The node can be a good recommender, can be said to be honest or an accurate accuser. On the contrary, its trustworthiness evaluation based on its actual networks’ operations may differ. For instance, nodes that are in zone 1 are said to be untrustworthy because of very poor network operations. For the nodes in zone 2, their trustworthiness will be undecided or uncertain, which may be as a result of limited first-hand knowledge of its actual network operations. The nodes found in zone 3 could be classified as being trustworthy with regards to their recommendations about other nodes and with regards to their actual network operations. Examples of nodes in this category are cooperative and good nodes.

In the case of zones 4, 5, and 6, nodes found in those zones are said to have undecided or uncertain trustworthiness in terms of the accuracy of their recommendations, not enough information to help reach a decision. In terms of their network operations, nodes in zone 4 will be evaluated as untrustworthy because of their poor network operations. The nodes in zone 5 will be said to have undecided or uncertain trustworthiness. These nodes are most likely newcomers to the network or inactive, broken, or faulty nodes.

Lastly, nodes in zone 7, 8, and 9 are all said to be untrustworthy in terms of the poor quality and accuracy of their recommendations. In evaluating the nodes based on their actual network operations, nodes in zone 7 will be classified as untrustworthy. These nodes are mostly malicious, attackers or intruders. The nodes in zone 8 will have undecided or uncertain trustworthiness, while those in 9 will be classified as being trustworthy. Most nodes found in zone 8 and 9 could be called liars, malicious accusers, or bad recommenders.

A similar categorisation was carried out by Zouridaki et al. [19], but their approach was based on a node’s ability to forward data packets and make good recommendations. Different other scenarios such as when a node carries out an attack such as grey-hole attacks, black-hole attacks were not considered. Furthermore, the approach presented in this paper considers a node that is new to a network. Moreover, the mathematical analysis for the categorisation is based on Dirichlet distribution which is fully analysed in a later section. Thus, the two-dimensional view approach aims to effectively evaluate the trustworthiness of a node.

### 3.1. Behaviours: Friendly and Threat Models

To observe, understand, analyse and categorise the behaviours of nodes in the proposed model, various behaviours have been designed which are exhibited and monitored during network operations. To ascertain the continuity of the network in the presence of selfish and malicious nodes, one of the proposed node behaviours is a well-behaved node which always guarantees that all the packets destined for other nodes are forwarded as expected and never dropped as long as a valid route is available. The selfish and malicious nodes serve as threat models while the well-behaved node serves as the friendly model. The different models depicting the nodes behaviours exhibited during network operations are given as:

- Good node: Nodes in this category respond to all route requests as expected and ensure that all the data and control packets that are meant for other nodes are forwarded to the next-hop node or the recipient node if they are the last hop in the path.
- Periodically selfish node: A periodically selfish node acts selfishly at regular intervals. The nature of its behaviour is aimed at conserving its limited energy resources rather than malicious. With regards to contributing to the network operations, it periodically participates in route discovery processes by forwarding control packets for other nodes. This is because control packets are smaller in size than data packets and consume less energy during packet transmission. Whenever a data packet is presented to this node for onward transmission, the data packet is dropped. In this threat model, a node intermittently replies to the route requests. For instance, it drops 2 out of every 3 control packets it receives but drops every data packet it receives for forwarding is dropped. Nodes that forward data packets to this node may perceive the network link as broken when they don’t receive acknowledgements for the first set of data sent. The network link to this node is then deleted from their route entries, but after a while, the connection is re-added when the node participates in the route discovery process again.
- Low energy-constrained selfish node: Nodes in this category acts as good node during the first period of the network operations. At the later stage of the network operations, it acts as a periodically selfish node due to reduced energy levels.
- Grey-hole node: A grey-hole node advertises valid routes. This node responds to all route requests it receives, but it periodically drops the data packets that are meant to be forwarded to the next-hop nodes or the recipient node if it’s the last-hop in the routing path. This node carries out grey-hole attacks during network operations.
- Black-hole node: A black-hole advertises valid routes whenever a route is requested. For example, in the Dynamic Source Routing (DSR) protocol, a black-hole node in- creases the sequence number in the route reply packet to the highest number possible so that the source node sees it as the nearest node to the required destination. Further- more, it drops all the data packets that are meant for any other nodes continuously.
- Malicious Packet Modifiers: Nodes in this category modify packets sent to it before forwarding it to the next hop. Malicious packet modifiers may modify the destination address of a packet before rerouting it. This could lead to denial-of-service attacks if it targets a specific node. Malicious packet modifiers could also decrease the Time-to-Live (TTL) in received packets to an artificially low value before forwarding them. This act means that a packet with a reduced TTL value may never reach its intended destination.

The other behaviours are based on the accuracy of second-hand reputations a node provides about other nodes. This behavioural model is categorised into two groups. These groups are given as:

- Honest Node: Nodes in this category disseminate accurate second-hand reputations about other nodes that they have had interactions with in the past. This is aimed at computing an accurate total reputation of the nodes in the network. The dissemination of accurate second-hand reputations about other nodes in the network assists nodes with limited first-hand information about a target node to evaluate and decide if a node can be relied upon or not.
- Dishonest Node: Nodes in this category disseminate false or incorrect second-hand reputations about other nodes in the network. This may be either for badmouthing or for ballot-stuffing the target nodes. Badmouthing of a node is a case whereby false second-hand reputation causes the evaluated trustworthiness of a node to decrease, while ballot-stuffing is a situation whereby the false second-hand reputations cause the evaluated trustworthiness of a node to increase.

### 3.2. The Importance of the Friendly and Threat Models

The described friendly and threat models are essential to this research work. The behaviours exhibited by these nodes will aid in evaluating the performance of the proposed RTM model under various conditions and scenarios. Some of these behaviours are exhibited as a combination such as a good and honest node. This type of node is good in terms of continuous forwarding packets and honest in terms of the accuracy of the second-hand reputation it provides about other nodes. The goal of having threat models such as grey-hole, black-hole, periodically selfish nodes, and malicious modifiers is to determine the performance of the network under various attacks, and a combination of various behaviours such as low energy-constrained selfish and honest behaviours will aid in analysing the proposed two-dimensional view of a node’s network activities. The total trustworthiness evaluation of a node in the proposed model is based on a combination of a node’s active and passive network activities.

## 4. The Proposed Dirichlet Reputation and Trust Management System

The proposed Dirichlet Reputation and Trust Management System consists of a monitor, a reputation module, a trust module, and a reward and punitive module. The following assumptions are employed in our Dirichlet reputation and trust management model.

- Every node operates in a promiscuous mode, such that each node listens to every packet transmitted by its neighbours, even if the packet is not intended for the node.
- Each node in the network will exhibit two of the behaviours described in Section 3.1 to evaluate the proposed model.

**Figure 2:**
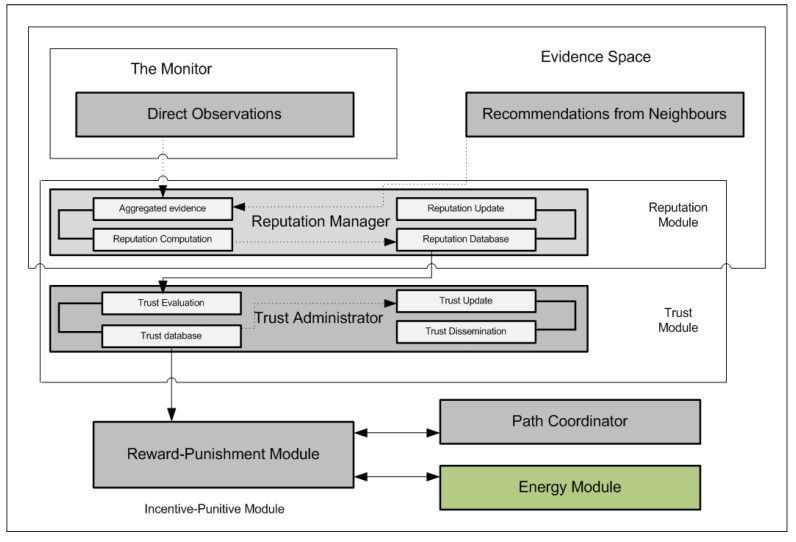
The schematic diagram of the Dirichlet Reputation and Trust Management System.

These assumptions are very important for the proposed model because we are more focused on the two-dimensional view approach that aims to effectively evaluate the trustworthiness of a node in the network. A depiction of the various core components of the proposed model is given in Figure 2. These core components make up the modules of the Dirichlet Reputation and Trust Management.

### 4.1. Monitoring Module (Monitor)

The monitoring module comprises entirely of the monitor class which is an essential part of the Dirichlet Reputation and Trust Management. The monitor is responsible for gathering the information used in evaluating the reputation and the corresponding trustworthiness of a node in the network. Failure by the monitor to observe the activities carried out by misbehaving nodes accurately may be very costly for the system. The monitor’s the monitor can observe different forms of behaviours that involve forwarding a packet, dropping packets, and maliciously modifying packets before forwarding. To ensure the viability of the monitoring processes, the monitor only observes the activities of nodes that are 1-hop away, and each node can carry out Omni-directional transmission. The monitor will be able to observe the following behaviours exhibited by nodes in the network.

- Successful packet forwarding
- Selective dropping is carried out by periodically selfish nodes.
- Malicious modification of packets before forwarding.
- Black-hole and grey-hole nodes that specialise in dropping data packets

### 4.2. Reputation Module

The reputation module comprises the reputation manager which is solely responsible for the evaluation of the various reputation values of nodes. The reputation manager evaluates the direct reputation of nodes based on the information derived from observing the nodes’ network activities. In a situation whereby the directly observed information is not sufficient to determine the reputation of a node, the reputation manager relies on second-hand reputation from neighbouring nodes to compute the total reputation value of a node. To avoid false recommendations (second-hand reputations) being used in computing the total reputation values of a target node, a deviation test is performed to determine the validity of the second-hand reputations. The result of the deviation test affects the corresponding trust values of the recommending nodes positively or negatively. The mathematical model used in evaluating the reputation of a node is the Dirichlet probability distribution. The Dirichlet probability distribution is used in evaluating the reputation of a node from the information obtained by the monitor when operating in the packet forwarding, packet dropping, and malicious modification of packet mode. The Dirichlet probability distribution is chosen over other distributions because it provides a sound and flexible platform suitable for designing a practical reputation system [34] Computing the reputation of nodes in MANETs using Dirichlet probability distribution was initially proposed in a paper by Chiejina et al. [4]. In terms of evaluating recommendations, it is useful in implementing recommendations with graded levels, i.e., very bad–bad–uncertain–good–very good. This enables nodes to evaluate recommendations effectively and integrate them in computing the total reputation of a node. The reputation manager passes the total computed reputation of a node to the trust module. The mathematical analysis of the Dirichlet probability distribution is in the next section.

#### 4.2.1. Modelling First-Hand Reputation Using Dirichlet Distribution

Nodes in the proposed model continuously observe the active network operations of their neighbours. For example, **N**_1_ and **N**_2_ are 1-hop away (**N**_1_ is the node carrying out the direct observation, while **N**_2_ is the node being observed). Let’s assume that **N**_1_ observes successful packet forwarding activities or possible dropping of packets by **N**_2_ in the network. Based on the various outcomes drawn from the independently observed behaviours, **N**_1_ assumes that the observed behaviours of **N**_2_ follow a probability pattern that can be derived using the Dirichlet distribution. The Dirichlet distribution can be employed in capturing a sequence of observations of k possible outcomes with k positive real parameters denoted as α_i_, where i = 1 (,… k), corresponds to one of the possible outcomes of the direct observations [34]. The sequence of observations of **N**_1_ and **N**_2_ comprises all the interactions that have occurred between **N**_1_ and **N**_2._

Let’s assume that the interactions can lead to three possible outcomes such as successful packet forwarding, dropping of packets, and malicious modification of packets. Let $\;\vec{p}$ represents the set of possible outcomes, i.e.,

(1) $$\;\vec{p}=\{p_{i}|1\leq i\leq 3\}$$

such that p_1_ represents the probability that **N**_2_ successfully forwards packets, p_2_ represents the probability that **N**_2_ drops packets and p_3_ represents the probability that **N**_2_ maliciously modifies packets before forwarding them. Let $\vec{\alpha }$ be a set of positive real parameters,

(2) $$\vec{\alpha }=\{\alpha_{i}|\;1\;\leq i\leq 3\}$$

The parameter α_i_ can be interpreted as the prior observation counts of the possible outcomes such that α_1_ represents the number of packets successfully forwarded by **N**_2_, α_2_ represents the number of successfully observed packet dropping by **N**_2_, and α_3_ represents the number of successfully observed malicious modification of packets by **N**_2_.

The Dirichlet probability density function (PDF) for the three possible expected outcomes and their corresponding counts can be defined as:

(3) $$f\left(\vec{p}|\vec{\alpha }\right)=\frac{\Gamma \left(\sum_{i=1}^{3}\alpha_{i}\right)}{\prod_{i=1}^{3}\Gamma \left(\alpha_{i}\right)}$$

Such that $p_{1},\ldots ,p_{3}\geq ,\alpha_{1},\ldots ,\alpha_{3}\geq \mathrm{and}$

(4) $$\sum_{i=1}^{3}p_{i}$$

The operator Γ represents the Gamma function. The expectation value of the K random variables can be defined as:

(5) $$E\left(\vec{p}|\vec{\alpha }\right)=\frac{\alpha_{i}}{\alpha_{0}}$$

where α_0_ is the sum of all α_i_, such that:

(6) $$\alpha_{0}=\sum_{i=1}^{3}\alpha_{i}$$

The Dirichlet distribution has only a k − 1 degree of freedom. This implies that deducing k − 1 probability variables and their density enable the deduction of the last probability variable and its density [34].

#### 4.2.2. Computing Direct Reputation Values Using Probability Expectation

As stated earlier, there are three possible events a monitoring node **N**_1_ can observe a target node **N**_2_ perform. These observable behaviours can be categorised as benevolent, selfish, and malicious. The observed behaviours in each category can be interpreted as ($\alpha_{1\;}\alpha_{2\;}\alpha_{3\;}$) which represent benevolent, selfish, malicious nature respectively. In Section 4.2.1, it was stated that the probability of monitoring node **N**_1_ to successfully observe the three defined events of a target node **N**_2_ is given by $\vec{p}$ = {p_i_|1 ≤ i ≤ 3}, which follows the defined Dirichlet distribution, i.e.,

Dir(α)~(p_1_, p_2_, p_3_) (7)

To evaluate the reputation values of **N**_2_ using the probability expectation of the Dirichlet distribution, let’s assume that **N**_1_ initially does not know **N**_2_, so cannot technically classify **N**_2_ into any of the three defined categories, i.e., [$\alpha_{1\;}\alpha_{2\;}\alpha_{3\;}$] = [0, 0, 0]. The values of [$\alpha_{1\;}\alpha_{2\;}\alpha_{3\;}$] are periodically updated at defined intervals, e.g., every 30 s which serves as the monitoring window. Let’s assume that **N**_1_ successfully captures a sequence of observations of **N**_2_ activities in the first interval of monitoring, say, 45 packets forwarded, 5 dropped packets, and 0 maliciously modified packets. This means that the values of $\left[\alpha_{1\;}\alpha_{2\;}\alpha_{3\;}\right]$ at t_1_ are [45, 5, 0]. So **N**_1_ will compute the posterior expected probability distribution of **N**_2_ which is equivalent to the reputation of node **N**_2_ based on its ability to deliver packet successfully as:

(8) $$R_{\mathrm{forwardingpackets}}=R_{\mathrm{fp}}=E\left[p1\right]=\frac{\alpha_{1}}{\alpha_{0}}=\frac{5}{45+5+0}=\frac{45}{50}=0.900$$

Similarly, the reputation of node **N**_2_ based on its ability to drop packets is computed as:

(9) $$R_{\mathrm{droppingpacketsts}}=R_{\mathrm{dp}}=E\left[p2\right]=\frac{\alpha_{2}}{\alpha_{0}}=\frac{5}{45+5+0}=\frac{5}{50}=0.100$$

And finally, the reputation of node **N**_2_ based on its ability to modify packets before forwarding is computed as:

(10) $$R_{\mathrm{maliciousmodificationofpackets}}=R_{\mathrm{mmp}}=E\left[p3\right]=\frac{\alpha_{3}}{\alpha_{0}}=\frac{0}{45+5+0}=\frac{0}{50}=0$$

As more and more evidence become readily available throughout subsequent monitoring intervals as illustrated in Table 1. Node **N**_1_ will update the evaluated reputation values accordingly.

(11) $$R_{\mathrm{fp}\;}=\frac{139}{160}=0.896,\;R_{\mathrm{dp}}=\frac{21}{160}=0.131,\;R_{\mathrm{mmp}}=\frac{0}{160}=0$$

We can therefore say that the reputation vector of **N**_2_ as observed and evaluated by **N**_1_ is a combination of three components based on the successfully observed behaviours. Thus, the directly observed and evaluated reputation vector of **N**_2_ is given as:

**R**_directreputation_ = ⟨**R**_fp_, **R**_dp_, **R**_mmp_⟩ = ⟨0.869, 0.131, 0.000⟩ (12)

The evaluated reputation values in Equation (12) show that we can establish the direct reputation of a node in the proposed model by observing three possible behaviours of nodes. $R_{\mathrm{fp}\;}$ serves as a positive experience the monitoring node derived from the monitored node (target node), while $R_{\mathrm{dp}}$ and $R_{\mathrm{mmp}}$ serves as negative experiences. The directly observed computed reputation values are used in evaluating the total reputation of a node if second-hand reputations from honest nodes are taken into account. It is quite important to state that modelling a node’s behaviour using the Dirichlet probability distribution with regards to the possible observable behaviours of a node can only be achieved if the node being observed participates in route discovery operations. If a node does not participate in route discovery operations, the node will not be presented with any packets for forwarding, so the possibility of dropping a packet or maliciously modifying the packet before forwarding will be zero.

#### 4.2.3. Second-Hand Reputations from Neighbours

Second-hand reputations from neighbouring nodes are employed in computing the total reputation of a node in the proposed model. This is important because a monitoring node may not have gathered enough evidence to truly ascertain if the target node is reliable or not. Mobile nodes that solely depend on their first-hand information before computing the reputation values of nodes in the network will only have a limited perspective about the network and may not be able to make accurate routing decisions. To evaluate the total reputation of a node, a monitoring node also relies on second-hand reputations from its neighbours. To incorporate second-hand reputations into computing the total reputation of a target node, a monitoring node sends a reputation request to its neighbours which contain the node-id of the target node. The reputation request contains three required values of a node’s network activities as a node’s reputation in terms of the forwarding packets for other nodes, its reputation in terms of dropped packets, and its reputation in terms of maliciously modified packets before forwarding. The recommending node only sends its evaluated firsthand reputation of the target node. To reduce the risk of flooding the network, the reputation request is only sent to neighbours that are 1-hop away excluding the node being monitored. When the monitoring node gets reputation replies from its neighbours, it filters genuine second-hand reputations from false second-hand reputations before computing the aggregated second-hand reputation values. This is done by carrying out a deviation test on all the second-hand reputation values it received from its neighbours. The result of the deviation test will affect the trust value of the recommending node positively or negatively. This method of carrying out a deviation test on received second-hand reputations is similar to the work carried out in [5,18,30,33]. For instance, if the direct reputation values (reputation vector) evaluated by a monitoring node **A** on a target node **B** is given as $R_{1}=\langle R_{{\mathrm{fp}}_{1}},R_{{\mathrm{dp}}_{1}},R_{{\mathrm{mmp}}_{1}}\rangle$ and the second-hand reputations of node **B** from node **C** are given as $R_{2}=\langle R_{{\mathrm{fp}}_{2}},R_{{\mathrm{dp}}_{2}},R_{{\mathrm{mmp}}_{2}}\rangle$ the deviation test can be evaluated based on the Euclidean distance of the reputation vectors as shown below;

(13) $$\sqrt{{\left(R_{{\mathrm{fp}}_{1}}-R_{{\mathrm{fp}}_{2}}\right)}^{2}+{\left(R_{{\mathrm{dp}}_{1}}-R_{{\mathrm{dp}}_{2}}\right)}^{2}+{\left(R_{{\mathrm{mmp}}_{1}}-R_{{\mathrm{mmp}}_{2}}\right)}^{2}}\geq \vartheta$$

ϑ is a positive constant and acts as the threshold validating second-hand reputations from other nodes. The value of the deviation constant, ϑ, was chosen as 0.3 for all the simulations carried out in this work. This was based on a comprehensive simulation study carried out on the directly computed values of several nodes (i.e., between 50 and 100 nodes) during this research works.

#### 4.2.4. Aggregated Second-Hand Reputations

**Figure 3:**
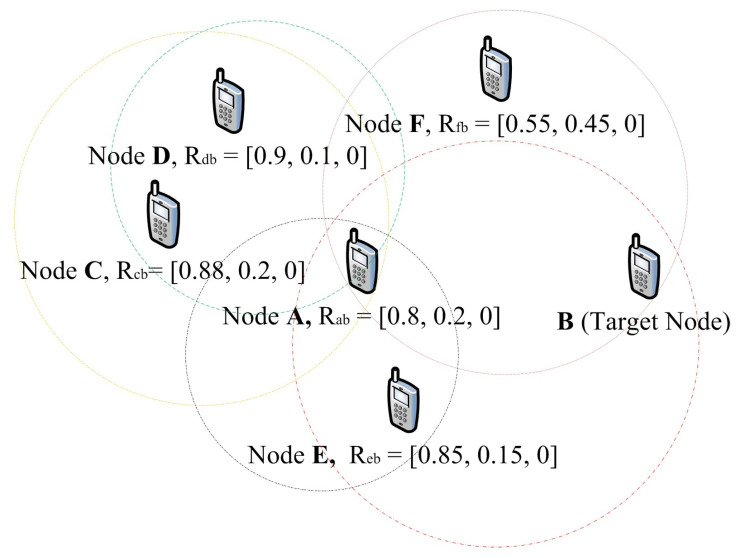
A typical scenario of second-hand reputations from neighbours.

Aggregated second-hand reputation is the summation of all valid second-hand reputations from a monitoring node’s neighbours about a target node. If node **A** has more than 1-hop neighbours that have had previous interactions or had completed successful observations of node **B** packet transmission activities. A reputation request is sent to all the neighbours. Every received second-hand reputation from the neighbours about a target node must undergo the deviation test before it is used in computing the aggregated second-hand reputation. A typical scenario is illustrated in Figure 3. If node **A** wants to compute the total reputation of a target node **B**. Node **A** sends a second-hand reputation request to all its neighbours that are 1-hop away, apart from node **B**. Node **A** has already evaluated the direct reputation vector $R_{\mathrm{ab}}$ of node **B** as

(14) $$\langle R_{\mathrm{fp}},R_{\mathrm{dp}},R_{\mathrm{mmp}}\rangle =\langle 0.900,0.100,0.000\rangle .\;$$

If all the 1-hop neighbours that have previously had an experience with node **B** send second-hand reputation replies to node **A**, it carries out the deviation test before computing the aggregated reputation of node **B**. Only second-hand reputations that are valid are incorporated into the aggregated reputation evaluation as seen in Table 2. If any of the received second-hand reputations from the neighbours fails the deviation test, as illustrated in Table 2. The second-hand reputation will not be used in computing the aggregated second-hand reputations of node **B**. Let $\vec{r_{b}}$ be the sum of all the valid (accurate) second-hand reputations from node **A** 1-hop neighbours about node **B** and let ${\vec{r_{b}}}^{i}$ be the individual second-hand reputations from each of the neighbours.

(15) $$\vec{r_{b}}=\sum_{i\in N}{\vec{r_{b}}}^{i}$$

From Table 2, the values of $\vec{r_{b}}$ be approximated as 〈0.880,0.120,0.000〉. This is the average of the individual reputation values representing the three different categories. The trust value of the node that provided the false second-hand reputation will be affected negatively, while that of nodes that provided valid secondhand reputations is affected positively. This trust value is based on the accuracy of the second-hand reputations a node provided.

#### 4.2.5. Computing the Total Reputation of Nodes

To evaluate the total reputation value of a target node, the aggregated second-hand reputations from the neighbours are combined with the computed direct reputations to get the total reputation values for the target node. For instance, the total reputation of node **A** about node **B** after a certain period t + 1 is given as:

(16) $$R_{\mathrm{ab}\left(t+1\right)}=\gamma R_{\mathrm{ab}\left(t\right)}+{\varphi r}_{\mathrm{cb}}$$

$R_{\mathrm{ab}\left(t+1\right)}$ is the updated reputation, $R_{\mathrm{ab}\left(t\right)}$ is the currently measured reputation values after subsequent monitoring intervals and ${\varphi r}_{\mathrm{cb}}$ is the second-hand reputation from neighbouring node C. The symbols γ and φ are positive weights that act as discount factors. The use of γ and φ in computing the total reputation of a node is to ensure that more weights are assigned to directly observed behaviours as compared to the aggregated second-hand reputations of nodes. Nodes in MANETs generally believe that their evaluated observed first-hand information about a target node is more accurate than second-hand reputations received from their neighbouring nodes. In the evaluation of the model the value of [γ, φ] = [0.8, 0.2]. The sum of γ and φ equals unity and γ is always greater than φ.

If node **A** has more than 1-hop neighbours that have had previous interactions with node **B** as illustrated in Section 4.2.4, the total reputation of node **A** about node **B** after a period t + 1 is computed using the equation:

(17) $$R_{\mathrm{ab}\left(t+1\right)}=\gamma R_{\mathrm{ab}\left(t\right)}+\varphi \left[\underset{i\in N,t}{\sum }{\vec{r_{b}}}^{i}\right]$$

and this can be simplified to

(18) $$R_{\mathrm{ab}\left(t+n\right)}=\gamma R_{\mathrm{ab}\left(t\right)}+{\varphi \;\vec{r}}_{b\left(t+1\right)}$$

where $\vec{r_{b\left(t\right)}}$ is the sum of all the valid second-hand reputations from node **A** 1-hop neigh-bours about node **B** during a given period t and ${\vec{r_{b}}}^{i}$ is the individual second-hand reputations aggregated from the equation given in 15. It can be stated that after n periods of time, the total reputation of node **A** about node **B** can be given as:

(19) $$R_{\mathrm{ab}\left(t+n\right)}=\gamma R_{\mathrm{ab}\left(t\right)}+{\varphi \;\vec{r}}_{b\left(t+n\right)}$$

The computed total reputation values of a node are used in determining the trustworthiness of a node using the novel candour two-dimensional trustworthiness evaluation technique.

## 5. Trust Module

The trust module computes and manages the trust evaluation of nodes in the network. Trustworthiness is a very essential property of nodes in the network because it helps to make informed routing decisions. Evaluating trustworthiness helps to determine which nodes are making positive contributions from nodes that are continuously displaying misbehaviours. Every node in the proposed model stores evaluated trust values in the database depicted in Figure 2. The novel candour two-dimensional trustworthiness evaluation of a node is a combination of two very important components which are the computed total reputation values of a node and the trust value in terms of its accuracy of the second-hand reputations it provides about other nodes. The former has been analysed in Section 4.2.5 and the latter will be discussed in the next subsection.

### 5.1. Trust Based on Accuracy of Second-Hand Reputations

To compute the trust value of a node with regards to the accuracy of its second-hand reputations about other nodes, the Bayesian approach is employed which means trust is expressed as having only two possible instances of behaviour of nodes, i.e., trustworthiness in terms of providing accurate second-hand reputations about other nodes and untrustworthiness in terms of providing inaccurate second-hand reputations about other nodes. This is different from the computation of the reputation of nodes using the Dirichlet distribution of which the behaviours of nodes are perceived to be benevolent (forwarding data packets), selfish (dropping data packets), and malicious (modifying packets before forwarding). Since the trust in terms of accuracy of second-hand reputations has two possible outcomes, employing the Beta distribution as a prior is adequate for the computational process. Mathematically, the Beta distribution is another form of the Dirichlet distribution with only two probability density function (pdf) shape parameters. The Beta distribution is conjugate. This means that a posterior probability will possess the same functional form as the prior. Hence, when the stored trust value of a node in terms of the accuracy of its second-hand reputations about other nodes is updated, the trust value will still follow the Beta distribution.

Let the trustworthiness of a node **A** about a target node **B** in terms of the accuracy of the second-hand reputations’ node **B** gives about other nodes be given as:

(20) $$T_{ab}∼\mathrm{Beta}\left(\varrho ,\chi \right)$$

where ϱ represents trustworthiness for accurate second-hand reputations and χ represents untrustworthiness for inaccurate second-hand reputations. At the onset of the network when a monitoring node has no prior knowledge of a target node’s ability to give accurate second- hand reputations, (ϱ,χ)=(1,1), which indicates a uniform distribution owing to the absence of prior knowledge. As second-hand reputations are received from the neighbouring nodes, the deviation test is computed for each set of received reputation values for the target node. As described in Section 4.2.3, using the Equation (13).

If the result of the deviation test is valid, the observed trust of the recommending node with regards to the accuracy of second-hand reputations about other nodes is updated positively. On the other hand, if the deviation test is invalid, the observed trust in terms of received inaccurate second-hand reputations is decreased.

Let ξ=1 when the deviation test is valid (i.e., when it succeeds), and let ξ=0 when the deviation test is invalid (unsuccessful), the new values of ϱ and χ is given as follows:

(21) ϱ=σϱ+ξ

(22) χ=σχ+(1−ξ)

where σ is the discount factor after a given period, and it’s such that σ∈[0,1] Equations (21) and (22) are similar to the equations employed by Buchegger and Boudec in [5]. For every deviation test executed whenever a second-hand reputation reply is received by the monitoring node, the stored trust data of the recommending nodes, i.e., (ϱ,χ) will be updated. The trust value for a node **B** as evaluated by a monitoring node **A** is determined by the expectation value of the Beta distribution. This is given by the equation below:

(23) $$\omega_{ab}∼E\left(\mathrm{Beta}\left(\varrho ,\chi \right)\right)=\frac{\varrho }{\varrho +\chi }$$

The computed expectation value $\omega_{ab}$ is used when evaluating the trustworthiness of a node in the network using the novel candour two-dimensional trustworthiness evaluation technique.

### 5.2. Trustworthiness of a Node

The candour two-dimensional trustworthiness evaluation of a node is determined by combining the total reputation values of a node and from the trust value in terms of the accuracy of second-hand reputations provided about other nodes. This decision is handled by the interaction decision-making module. The interaction decision-making module is responsible for deciding which nodes are trustworthy of carrying out reliable network operations. The decision-making process is briefly described as follows. Let’s assume that a monitoring node **A** wants to determine if a target node **B** is completely trustworthy in terms of its actual network operations (what it does) and what it says about other nodes. Node **A** relies on the computed total reputation values (total reputation vector) and the trust values in terms of the accuracy of second-hand reputations. Let’s define some very important thresholds 〈f,s,m〉 which serves as the expressions for tolerance in terms of reputation for forwarding packets for others, selfishly dropping packets, and malicious modification of packets before forwarding respectively. Furthermore, we also define $\tau^{t}$ as the threshold for the trustworthiness with regards to the accuracy for the second-hand reputations. For node **A** to classify node **B** as a totally trustworthy node with regards to its overall network behaviours’, the following conditions must be met.

With regards to its behaviours i.e., forwarding packets, dropping packets

$$\{\begin{array}{c} \mathrm{benevolent}\;\;\mathrm{if}\;\;\;\;\;\;\;\;\;\;\;\;R_{{\mathrm{fp}}_{T}}\;\geq \;f\;\\ \mathrm{selfish}\;\;\mathrm{if}\;\;\;\;\;\;\;\;\;\;\;\;\;\;\;\;\;\;\;R_{{\mathrm{dp}}_{T}}\;\geq \;s\\ \mathrm{malicious}\;\;\mathrm{if}\;\;\;\;\;\;\;\;\;\;\;\;\;R_{{\mathrm{mnp}}_{T}}\;\geq \;m \end{array}$$

and its trustworthiness with regards to the accuracy of its second-hand reputations as

$$\{\begin{array}{c} \mathrm{honest}\;\;\mathrm{if}\;\;\;\;\;\;\;\;\omega_{ab}\geq \tau^{t}\\ \mathrm{dishonest}\;\;\mathrm{if}\;\;\;\;\omega_{ab}<\tau^{t} \end{array}$$

It has already been established that the sum of the directly observed individual reputation values of a target such as a node **B**, $\langle R_{\mathrm{fp}},R_{\mathrm{dp}},R_{\mathrm{mmp}}\rangle$ by a monitoring node **A** as equals 1. Consequently, it is expected that the sum of the evaluated total reputation vectors $R_{{\mathrm{Total}}_{\mathrm{fp}}},R_{{\mathrm{Total}}_{\mathrm{dp}}}\;\mathrm{and}\;R_{{\mathrm{Total}}_{\mathrm{mmp}}}$, must be 1 as along as the second-hand reputations are accurate.

Therefore, for node **A** to be totally trustworthy, its total reputation with regards to its behaviour must be classified as benevolent and its trust value with regards to the accuracy of second-hand reputations must be classified as honest. Nodes that fall into the category of being totally trustworthy with regards to their entire network operations are permitted to continue their positive network contributions i.e.,

$$\left[R_{{\mathrm{Total}}_{\mathrm{fp}}},\omega_{ab}\right]=\left[\mathrm{benevolent},\mathrm{honest}\right]$$

**Figure 4:**
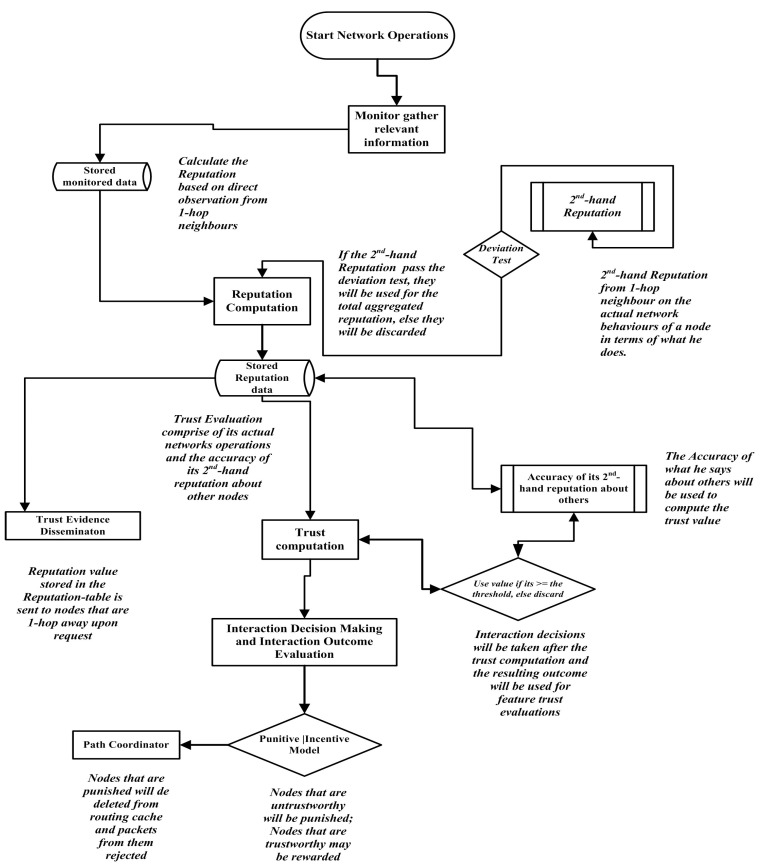
The flow chart for the Dirichlet Reputation and Trust Computation in the TRM Model.

On the other hand, nodes that are categorised as being untrustworthy i.e.,

$$\left[R_{{\mathrm{Total}}_{\mathrm{mmp}}},\omega_{ab}\right]=\left[\mathrm{malicious},\mathrm{dishonest}\right]$$

are punished. Nodes in this category are isolated by ensuring that the entire route request that they generate are ignored. All the paths containing these nodes are deleted from the route cache. Finally, a special case of a node being classified as **selfish** but **honest** with regards to the accuracy of its second-hand reputations is handled during the trustworthiness evaluation process. Nodes in this category are not totally isolated from the network. Selfish behaviour displayed by a node in the network may be triggered by a node’s physical properties (loss of battery power, being overwhelmed by route, and forwarding requests). It may also be a resolute attempt to conserve its resources (battery and computing resources), or a random failure. On the other hand, misbehaving nodes reduce the reliability of the network. These malicious nodes misroute, modify, or inject packets (making them a part of a different data transfer). These nodes are mostly interested in attacking and damaging the network. Malicious nodes generally lower the security and integrity of the network traffic. The interactions between the monitoring, reputation and trust modules can be seen in Figure 4. Figure 4 presents an overview of the entire working of the proposed system.

## 6. Implementation and Simulations

We designed and programmed the modules described above using C++ and implemented the various classes to work with existing NS-2.34 modules [35]. Several modifications were also carried out on existing NS-2.34 modules to incorporate the various required node behaviours and the overall functionality of the proposed Dirichlet reputation and trust management model. Ad Hoc On-Demand Distance Vector (AODV) was used as the routing protocol to verify the functionalities of the proposed model. Exhaustive simulations were also carried out, averaging 10 simulations for each specified scenario. This was aimed at replicating ten networks with different topologies. Seven of the ten nodes displayed good behaviours with regards to forwarding data packets, while the other three nodes were designated to act as black hole node, grey-hole, and periodically selfish node respectively. ϑ is the deviation constant for verifying received a second-hand reputation for a target node. γ and  φ are the weights assigned to the directly computed (first-hand) reputation and second-hand reputation values respectively. These were used for computing the total reputation values of nodes. σ is the aging factor assigned to computed trust values. All these general parameters used are mentioned in Table 3.

## 7. Results and Analysis

This section presents the simulation results showing the evaluated reputation and trust values that a node computes after successful observations of its neighbours’ activities are analysed. Comprehensive analyses of the computed direct reputation values (first-hand), the second-hand reputations, and the total reputation of nodes will aid in understanding nodes’ behaviours in a MANET without bias such that both negative and positive behaviours exhibited by a node are reflected (observed) from the evaluated reputation and trust values.

### 7.1. Evaluation of Directly Computed (First-Hand) Reputation

**Figure 5:**
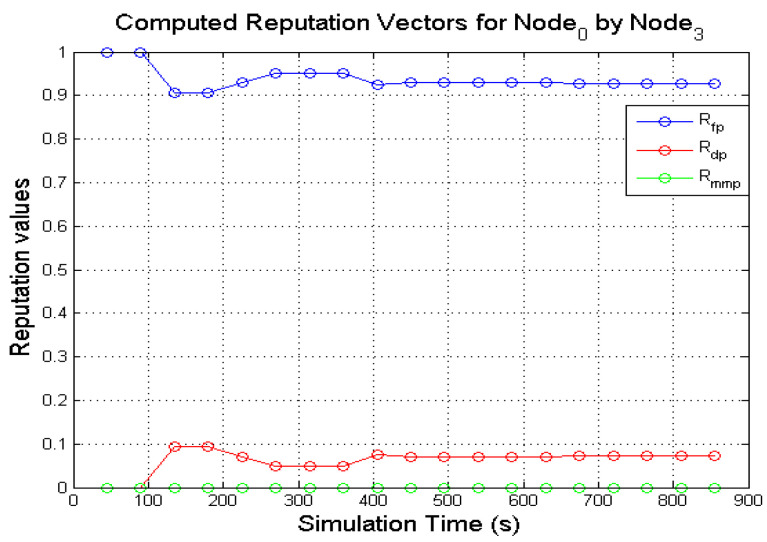
Reputation values for a good node (**N**_0_) calculated by another good (**N**_3_).

**Figure 6:**
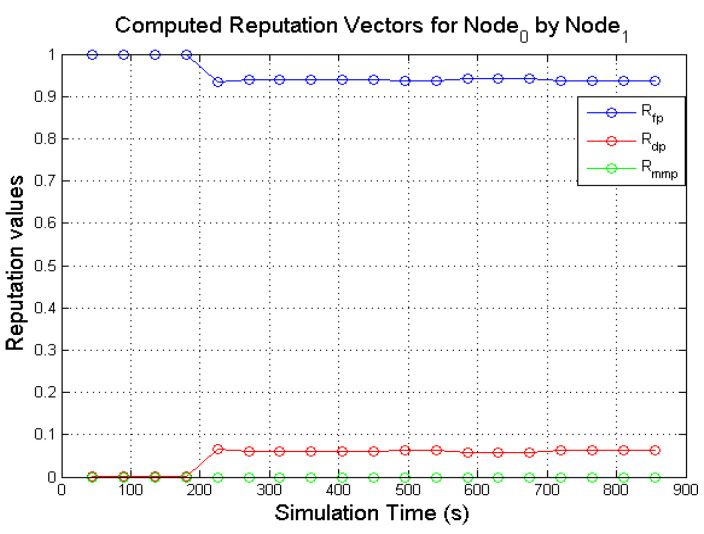
Reputation values for a good node (**N**_0_) calculated by periodically selfish node (**N**_1_).

The expectation values of the Dirichlet distribution were used in computing the various reputation values of nodes in the network. Figure 5 and Figure 6 presents the computed reputation vector of a target **N**_0_ by two monitoring nodes (**N**_1_ and **N**_3_). The designated behaviours of **N**_0_, **N**_1,_ and **N**_3_ are shown in Table 4. The *x*-axis represents the simulation

It is expected that a good node such as **N**_0_ will continuously forward every data packet that is presented to it subject to a valid route being available. The direct reputation vector of a node in the proposed model, given $R_{\mathrm{directreputation}}=\langle R_{\mathrm{fp}},R_{\mathrm{dp}},R_{\mathrm{mmp}}\rangle$, is a combination of three components as explained in Section 4.2.2. $\langle R_{\mathrm{fp}},R_{\mathrm{dp}},R_{\mathrm{mmp}}\rangle$ represents the 3-tuples $\langle R_{\mathrm{Benevolent}},R_{\mathrm{Selfish}},R_{\mathrm{Malicious}}\rangle .$

As illustrated in Figure 5 and Figure 6, the target node (**N**_0_) is observed as forwarding data packets continuously by **N**_1_ and **N**_3_ which is reflected on the computed values of $R_{\mathrm{fp}}$. It can also be observed that both monitoring nodes (**N**_1_ and **N**_3_) computed respective values for $R_{\mathrm{dp}}$ from **N**_0_ activities. Although **N**_0_ is selected to display benevolent behaviours during the simulation. The computed $R_{\mathrm{dp}}$ was as a result of incorrect observation outcomes. Further investigations from analysing the NS2 trace files show that the few packets dropped by **N**_0_ was as a result of buffer overflow of the packet queue. The packet queue holds packets that are meant for forwarding. It has a maximum number of packets it holds (50 packets in the simulations) while the forwarding node sources for the required path for the packets from the route cache. As more packets are received for forwarding, a good node may unintentionally drop the packets due to buffer overflow. Additionally, some of the dropped packets were as a result of packet expiration caused by queue time out or when packet TTL (Time-To-Live) reaches zero. Every packet has a limit it can stay in a queue before it times out. If the required path is not found before the queue times out, that packet may be dropped. These various packet drops may result in a good node being perceived as displaying selfish behaviour. However, since computing the direct reputation values are executed after monitoring is completed in the given monitoring interval as described in Section 4.2.2. As long as $R_{\mathrm{dp}}$ does not exceed the defined threshold, the value of $R_{\mathrm{dp}}$ is negligible. On the other hand, no value for $R_{\mathrm{mmp}}$ was computed all through the various monitoring windows which were expected.

**Figure 7:**
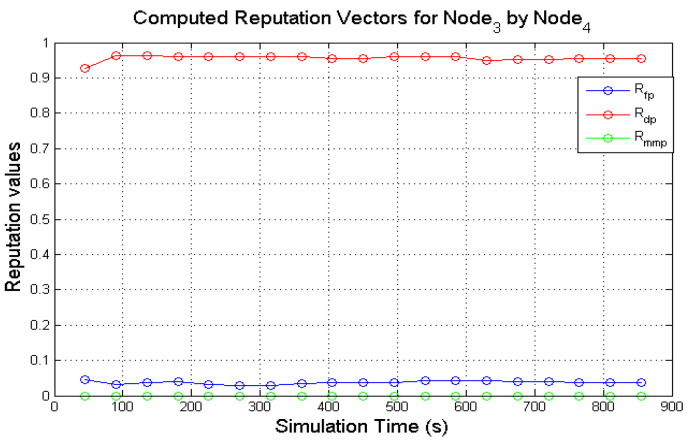
Reputation values for a periodically selfish node (**N**_3_) calculated by good node (**N**_4_).

**Figure 8:**
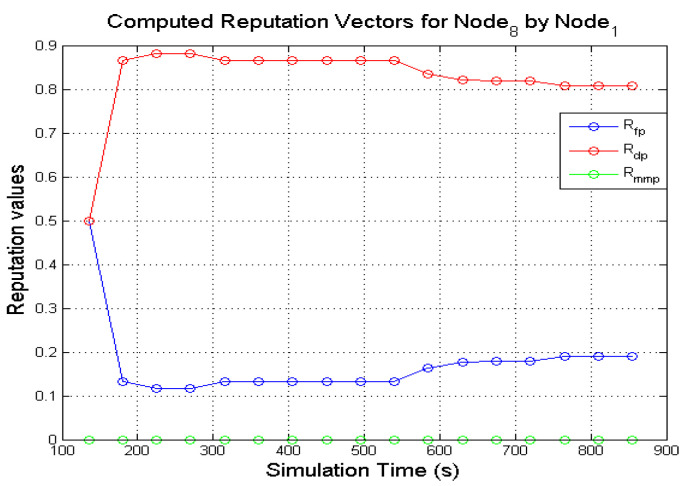
Reputation values for grey-hole node (**N**_8_) calculated by good node (**N**_1_).

**Figure 9:**
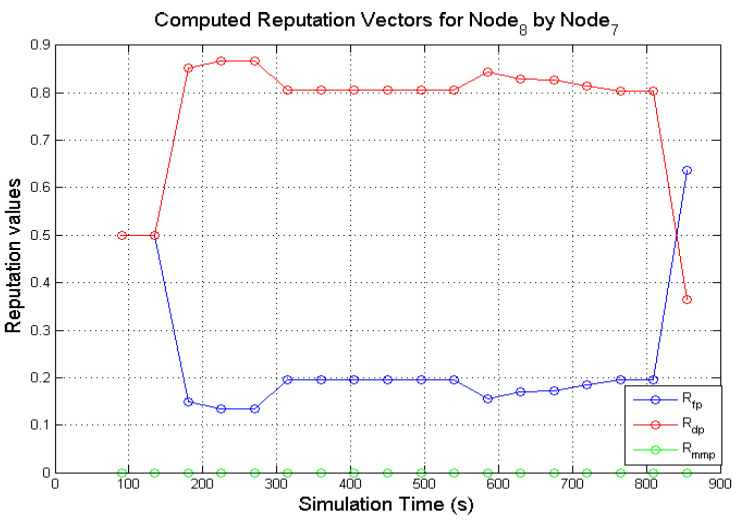
Reputation values for grey-hole node (**N**_8_) calculated by good node (**N**_7_).

**Figure 10:**
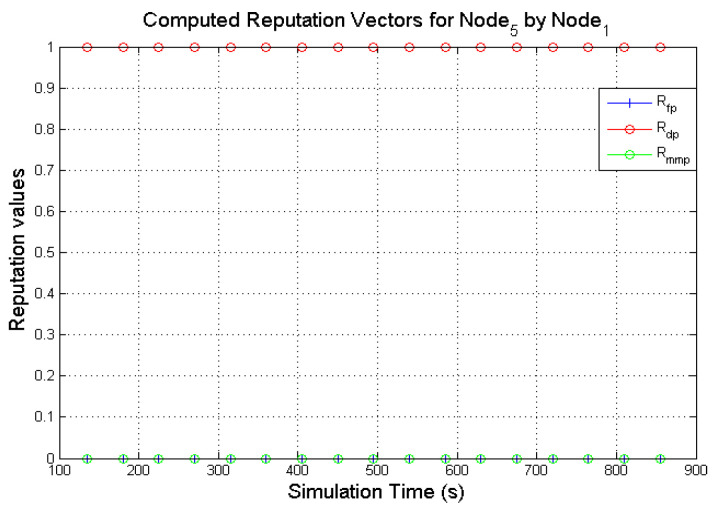
Reputation values for grey-hole node (**N**_5_) calculated by good node (**N**_1_).

The computed values of **N**_0_ by **N**_1_ and **N**_3_ demonstrate that the expectation values of the Dirichlet distribution are a viable mathematical solution to determine the reputation values of nodes in a network. Before this notion was fully established, the computed direct reputation values of three other behaviours (the three misbehaviours: periodically selfish node, grey-hole node and black-hole node) were also analysed as seen in Figure 7, Figure 8, Figure 9 and Figure 10.

Figure 7 presents the computed reputation values of **N**_3_ exhibiting a periodically selfish behaviour. Due to its behavioural nature **N**_3_ rarely responds to route requests which means that data packets are scarcely presented to it for forwarding. Any data that it receives as a result of participating in route discovery processes are dropped. In Figure 7 it is observed that the values $R_{\mathrm{fp}}$ is lower than 0.1 through the course of the recorded simulation time while $R_{\mathrm{dp}}$ is higher than 0.9. This indicates that **N**_3_ displayed the expected behaviour and the computed reputation values using the expectation of the Dirichlet distribution can model this behaviour.

Similarly, Figure 8 and Figure 9 presents the computed direct reputation values of **N**_8_ by **N**_1_ and **N**_8_ by **N**_7_. The behaviours of a grey-hole node are sometimes difficult to perceive from monitoring because of the deceptive nature of the node. A grey-hole node occasionally forwards data packets, but it can easily switch behaviours by dropping data packets maliciously. As shown in Figure 8, after the first window of observation, **N**_1_ could have been observed dropping and forwarding an equal number of packets which is reflected in the computed reputation values ($R_{\mathrm{fp}}$ and $R_{\mathrm{dp}}$ was computed as 0.5). Subsequent computed reputation values show $R_{\mathrm{fp}}$ increasing to 0.9 while $R_{\mathrm{dp}}$ decreased to 0.1. As more successful observation windows are completed, the deceptive nature of **N**_8_ is reflected in the computed reputation values as observed in Figure 8. The same trend is also observed from the computed reputation values carried out by **N**_7_ after observing the activities of **N**_8_ as shown in Figure 9. An interesting feature of the graphs in Figure 9 is the gradual decrease and increase in the computed values of $R_{\mathrm{dp}}$, and the reverse is observed in the values of $R_{\mathrm{fp}}$. After the first observation window was completed, the computed reputation values $\langle R_{\mathrm{fp}},R_{\mathrm{dp}},R_{\mathrm{mmp}}\rangle$, were ⟨0.5, 0.5, 0⟩. Subsequent computations show that the values of $R_{\mathrm{fp}}$, $R_{\mathrm{dp}}$ varied as the simulations progressed. This sort of behaviour could be difficult for the monitoring node to capture which is reflected in the computed values of $R_{\mathrm{fp}}$ and $R_{\mathrm{dp}}$ as the simulation time reached the 850 s mark (the value of $R_{\mathrm{fp}}$ registered a sharp decline, while $R_{\mathrm{dp}}$ registered a sudden increase) as observed in Figure 9. Thus, the incorporation of genuine second-hand reputations from neighbouring nodes could assist a monitoring node with further information about a target node. The decision to incorporate second-hand reputations.

### 7.2. Incorporating Accurate Second-Hand Reputations

Genuine aggregated second-hand reputations from 1-hop neighbours can be incorporated into the directly computed reputation values to get the total reputation values for a node being monitored. Honest second-hand reputations from 1-hop neighbours could benefit a monitoring node on a grey-hole target using the following examples in Figure 8 and Figure 9 in Section 7.1. Due to the changing behaviours of **N**_8_, **N**_7_ could find it difficult to reach a decision about the behaviours of a grey-hole node (**N**_8_) based on the directly computed reputation values. Assuming **N**_1_ provides genuine second-hand reputations about other nodes. If **N**_7_ and **N**_1_ are 1-hop neighbours, **N**_7_ can send a reputation request to **N**_1_ about **N**_8_ during the simulations. From Figure 8 it can be observed that **N**_1_ computed reputation values for **N**_8_ from approximately 135 s of the simulation time. If **N**_7_ sends a reputation request about **N**_8_ to **N**_1_, the values contained in the reputation reply will pass the deviation test that is performed to ensure that second-hand reputations are valid. The genuine second-hand reputations can be incorporated in calculating the total reputation of **N**_8_.

**Figure 11:**
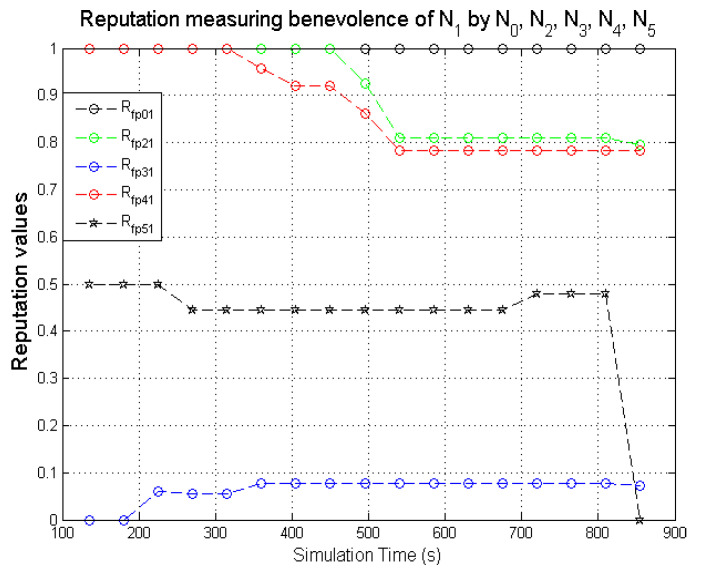
The reputation values ($R_{\mathrm{fp}}$) for **N**_1_ computed by **N**_0_, **N**_2_, **N**_3_, **N**_4_, & **N**_5_.

**Figure 12:**
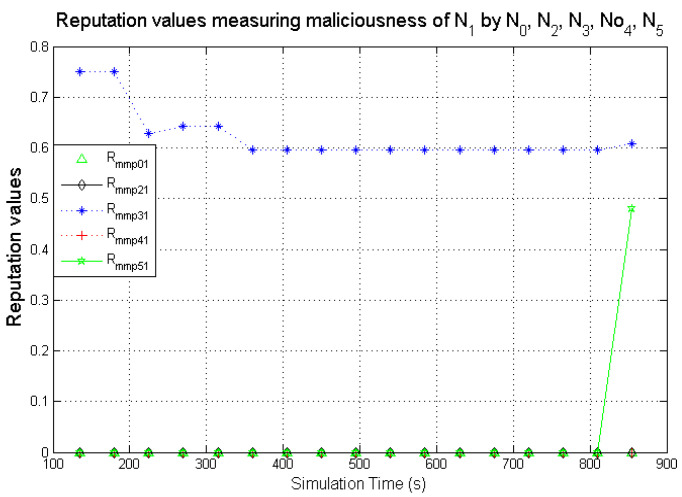
The reputation values ($R_{\mathrm{mmp}}$) for **N**_1_ computed by **N**_0_, **N**_2_, **N**_3_, **N**_4_, & **N**_5_.

**Figure 13:**
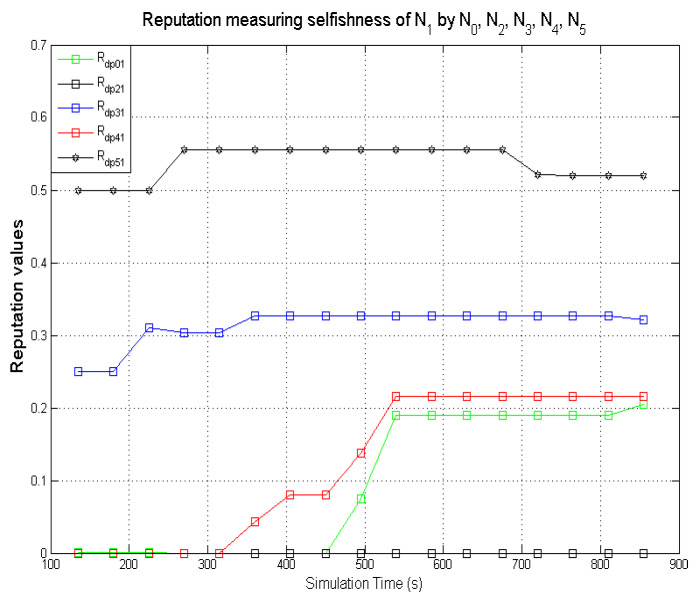
The reputation values ($R_{\mathrm{dp}}$) for **N**_1_ computed by **N**_0_, **N**_2_, **N**_3_, **N**_4_, & **N**_5_.

The graphs present simulation results showing the comparison of computed direct reputation values and second-hand reputations from neighbouring nodes. A target node **N**_1_ (exhibiting benevolent behaviour) was monitored by **N**_0_. **N**_2_, **N**_3_, **N**_4_, and **N**_5_ are 1-hop neighbours to **N**_0_. The behaviours displayed by the various nodes during the simulations are given in Table 5. The second-hand reputations from (**N**_2_, …, **N**_5_) represent benevolence, selfishness, and maliciousness. **N**_3_ and **N**_5_ are designated to act as dishonest nodes so the second-hand reputation values they passed on to **N**_0_ are inaccurate (the inaccurate second-hand reputations from dishonest nodes are generated such that it reflects a different nature from the behaviour being observed). As observed in Figure 11, Figure 12 and Figure 13, there are significant differences in the respective second-hand reputation values from **N**_3_ and **N**_5_ when compared to the reputation values computed by **N**_0_, **N**_2_, and **N**_4_. When the deviation test is carried out on the received second-hand reputation values at the various time intervals, the values from **N**_3_ and **N**_5_ will always fail the test because for each computed case the result will be higher than ϑ which represents the threshold, and the result must not exceed this value for it to be valid as evaluated in Section 5.1.

For node **N**_0_ to compute the total reputation values of the target node **N**_1_, **N**_0_ aggregates the genuine second-hand reputations from nodes **N**_2_ and **N**_4_ before computing it with its own directly measured reputation values to get the total reputation values for **N**_1_.

Their subsequent trustworthiness with regards to the accuracy of second-hand reputations is updated positively.

One of the benefits of incorporating second-hand reputations from genuine neighbours is that it could speed up the process of ascertaining the trustworthiness of a target node.

Furthermore, accurate second-hand reputations could also help a monitoring node to decide if its neighbouring nodes are honest.

### 7.3. Evaluation of the Two-Dimensional Trustworthiness of Nodes

Evaluating the total reputation values of a target node requires the combination of the directly computed reputation values and the aggregated accurate second-hand reputations from honest nodes. In the last section, it was established through analysing simulation results that the Dirichlet distribution is effective in modelling the behaviours of nodes. One important aspect of this research work is to determine how the observed and evaluated optimal weight of a target node can be used in determining the trustworthiness of a node. The optimal weights in this case are the most favourable evaluated total reputation and trust values observed by a monitoring node before establishing the trustworthiness of a target node in the proposed model. This requires analyses of various computed total reputation values of different target nodes and the trust values of the nodes based on the accuracy of the second-hand reputations it provides about other nodes. To achieve this goal, simulations were carried out using the parameters given in Table 6.

The simulations were carried out using a fixed network of 20 nodes. 20 different scenarios representing 20 different network topologies were randomly generated which was aimed at replicating real live ad hoc networks. One important factor about the simulations carried out is to get the right proportion of the node behaviours mixture with regards to the benevolent, selfish, and malicious nature of nodes. For sustainable and effective simulations, it is important to ensure that nodes that will continue to forward packets for other nodes are readily available. This ensures that the network data transfer process is not halted as the simulation progresses. Having more good nodes in the network ensures data availability, increases the network lifetime and improves the probability that data packets from the source will get to the desired destinations. With regards to second-hand reputations from nodes neighbouring nodes, there is a need to have a balance in the proportion of honest recommenders and liars. A scenario whereby there are only liars in the network will defeat the goal of evaluating the trustworthiness of a target node based on what it does with regard to packets and what it says about other nodes.

**Figure 14:**
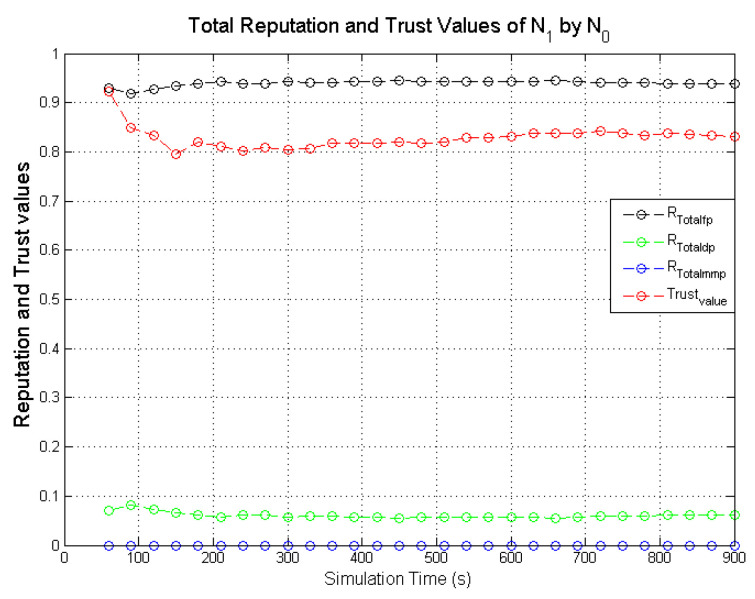
Nodes **N**_1_ and **N**_0_ are both benevolent and honest.

**Figure 15:**
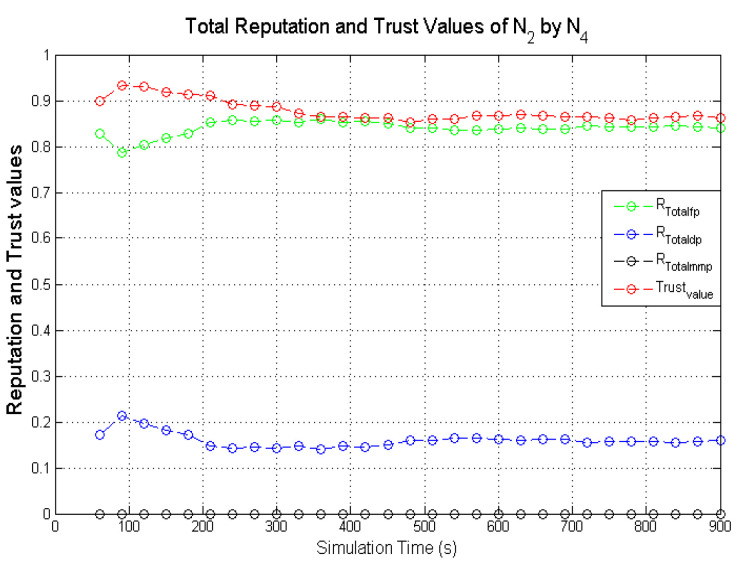
Nodes **N**_2_ and **N**_4_ are both benevolent and honest.

**Figure 16:**
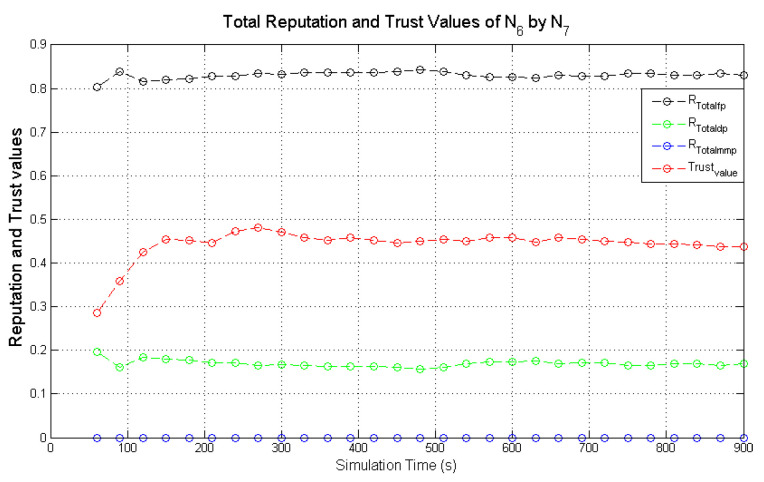
**N**_6_ is benevolent and dishonest, **N**_7_ is benevolent and honest.

**Figure 17:**
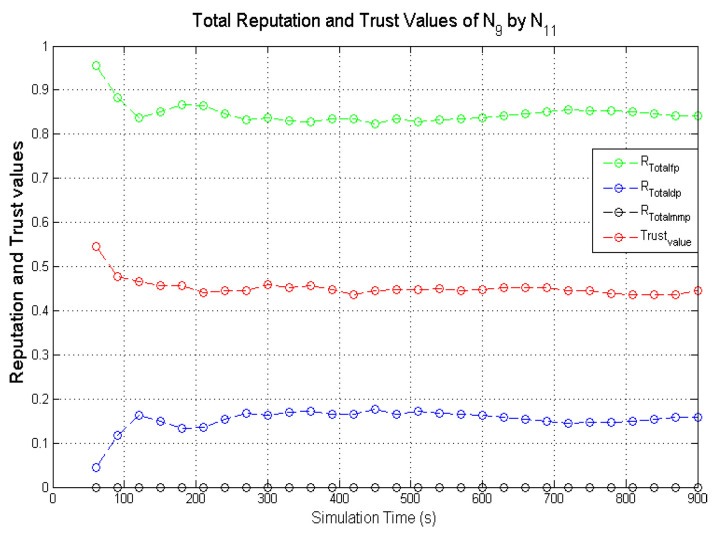
**N**_9_ is benevolent and dishonest, **N**_11_ is selfish and honest.

**Figure 18:**
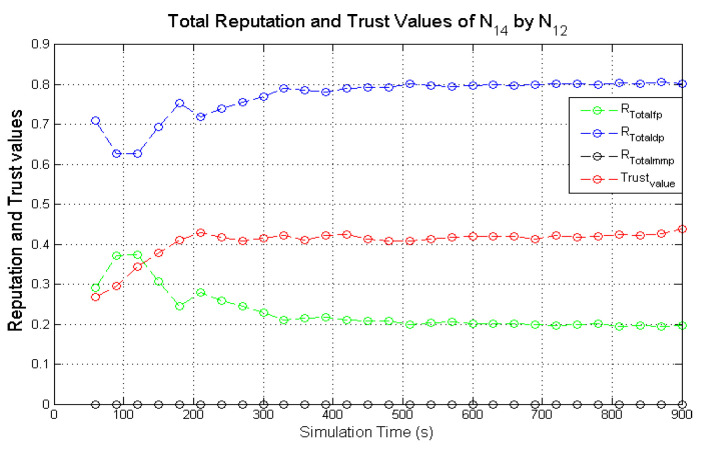
Nodes **N**_12_ and **N**_14_ are both selfish and dishonest.

**Figure 19:**
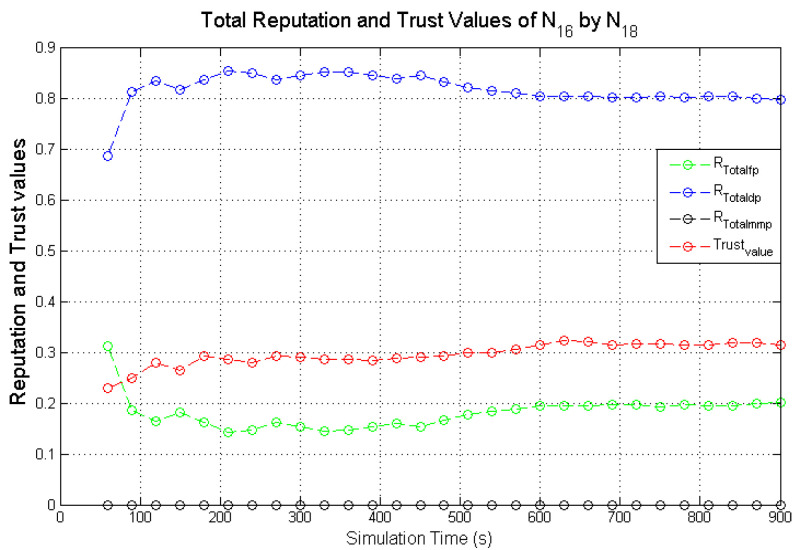
**N**_16_ is selfish and dishonest, **N**_18_ is benevolent and honest.

Observing the two-dimensional view of a node’s network activities presents the novel candour two-dimensional trustworthiness evaluation technique to determine the trustworthiness of a node based on two important qualities as proposed at the onset of this research work. That is a target node’s total reputation which measures its ability to forward packets and its honesty which measures its ability to provide genuine second-hand reputations. From the computed values of the total reputation and trust of **N**_1_ and **N**_2_ as observed in Figure 14 and Figure 15, the values for the set of thresholds ⟨f, s, m⟩ defined in Section 5.2 can be derived. However, before specifying the threshold values a target node must attain or not exceed before it can be categorised as being benevolent, selfish, or malicious. An overview of how other behaviours were observed and evaluated was also analysed. Figure 16 and Figure 17 show the graphs of the computed total reputation and the trust values of nodes **N**_6_ and **N**_11_ designated to act as good nodes with regards to packet forwarding and dishonest nodes with regards to second-hand reputations, they provide about other nodes. It is expected that the computed total reputation values of **N**_6_ and **N**_11_ ($R_{\mathrm{Totalfp}}$) would increase as the network operation progresses. This is mainly because packets presented are forwarded to the desired destination or the next hop as the case may be. In terms of the reputation values measuring the selfishness and the malicious of nodes **N**_6_ and **N**_9_, as observed in Figure 16 and Figure 17, the computed values of $R_{\mathrm{Totaldp}}$ are within the range of (0.02, 0.2) while that of $R_{\mathrm{Totalmmp}}$ is zero all through the observed network operation The computed values of the 3-tuples $\langle R_{\mathrm{Totalfp}},R_{\mathrm{Totaldp}},R_{\mathrm{Totalmmp}}\rangle$ confirms that nodes **N**_6_ and **N**_9_ exhibited the expected behaviours as observed and modelled by **N**_7_ and **N**_11_ using the Dirichlet distribution and second-hand reputations from neighbours. On the other hand, the trust values of both nodes are evaluated to be within the range of (0.46, 0.54). When compared to the computed trust values of **N**_1_ and **N**_2_ as observed in Figure 14 and Figure 15, **N**_1_ and **N**_2_ performed far much better than **N**_6_ and **N**_9_. Given these variations in the computed trust values, it is fair and appropriate to ensure that when categorising the four nodes **N**_1_, **N**_2_, **N**_6,_ and **N**_9_, a unique distinction can be drawn as to which nodes are good enough to be called totally trustworthy. This distinction is not required if the nodes behave badly such as being selfish and disseminating false second-hand reputations as seen in Figure 18 and Figure 19.

As observed in Figure 18 and Figure 19, the computed total reputation values $R_{\mathrm{Totaldp}}$ which measures the selfishness of nodes **N**_14_ and **N**_16_ were evaluated to be within the range of (0.62, 0.8) and (0.68, 0.86) respectively. This reflects the expected designated behaviours of nodes **N**_14_ and **N**_16_ and it confirms that the two monitoring nodes **N**_12_ and **N**_18_ successfully observed their packet forwarding activities. The computed values of $R_{\mathrm{Totalfp}}$ and $R_{\mathrm{Totalmmp}}$ also reflects the behaviours of both nodes. Similarly, being dishonest nodes, it is expected that observed computed trust values of nodes **N**_14_ and **N**_16_ will be below all through the simulations when compared to honest nodes likes nodes **N**_1_ and **N**_2_ as observed in Figure 18 and Figure 19. To ascertain the total trustworthiness of a node using the candour two-dimensional trustworthiness evaluation technique, nodes **N**_14_ and **N**_16_ will be categorised as being totally untrustworthy, which is reflected in the computed total reputation and trust values as observed and evaluated by nodes **N**_12_ and **N**_18_. If punitive measures were to be taken against nodes that fall under this category, it will be justified if nodes **N**_14_ and **N**_16_ are denied the limited available resources.

## 8. Discussions

**Figure 20:**
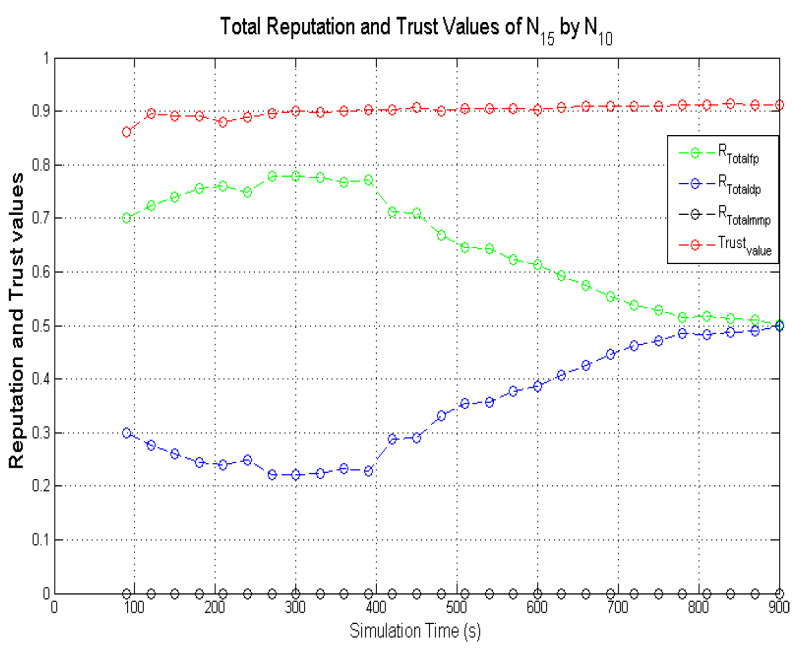
**N**_15_ is low energy-constrained selfish and honest, **N**_10_ is benevolent and honest.

**Figure 21:**
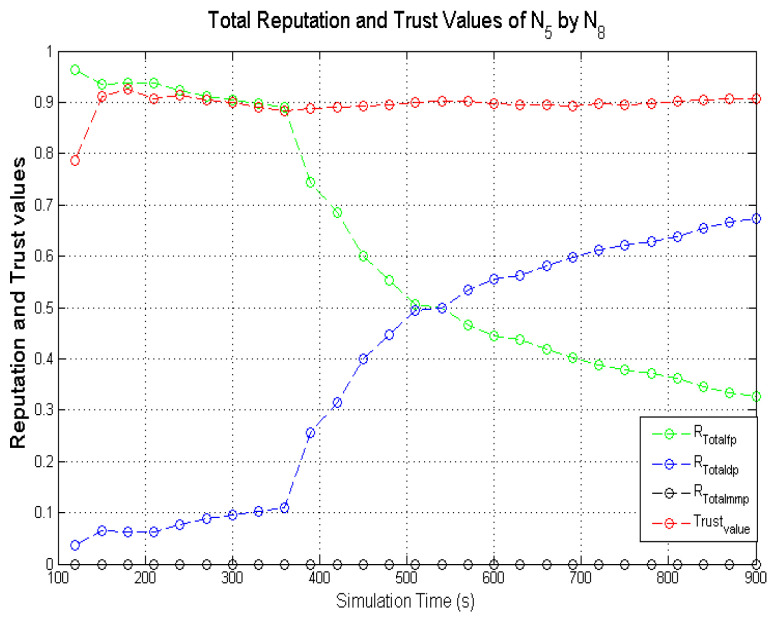
**N**_5_ is low energy-constrained selfish and honest, **N**_8_ is benevolent and honest.

In the process of evaluating the trustworthiness of a node, the candour concept must be preserved. For instance, if a target node was initially perceived as being benevolent due to the observed packet forwarding activities and honest with regards to accuracy of second-hand reputation (second-hand reputations), the target node will be categorised as being totally trustworthy if its computed total reputation and trust values meet the required thresholds. If the situation changes with regards to its packet forwarding activities as a result of reduced energy levels after subsequent monitoring intervals (observation windows) are completed, this node may be categorised as being selfish if the computed $R_{\mathrm{Totalfp}}$ value falls below the threshold while that of $R_{\mathrm{Totaldp}}$ increases. Typical scenarios are illustrated in Figure 20 and Figure 21, which present the computed total reputation and trust values of nodes **N**_5_ and **N**_15_. **N**_5_ and **N**_15_ exhibited more benevolent behaviours than selfish behaviours in the first part of the simulations and later changed their behaviours to more selfish than benevolent as their energy levels dropped to a set threshold. The observed weights are the computed total reputation values and the trust values such as: $\langle R_{\mathrm{Totalfp}},R_{\mathrm{Totaldp}},R_{\mathrm{Totalmmp}}\rangle$, and $\omega_{ab}$.

Further analyses of the computed values in Figure 21 show that the computed total reputation value $R_{\mathrm{Totalfp}}$ of node **N**_5_ dropped below 0.5 after the 570s. In this scenario, if node **N**_5_ is categorised as selfish and penalised afterward, the monitoring node may be justified as long as the penalty does not involve total isolation of node **N**_5_ from the network due to its continuous dissemination of genuine second-hand reputations. For candour which represents fairness to be incorporated into the categorisation of nodes the optimal weights of the set thresholds which determine the trustworthiness of nodes is specified within a given range such that $\langle R_{\mathrm{Totalfp}},R_{\mathrm{Totaldp}},R_{\mathrm{Totalmmp}}\rangle$ = ⟨(0.5 → 0.75),(0.50 → 0.25), 0⟩. This argument can be further justified when the computed total reputation and trust values of node **N**_15_ depicted in Figure 20 are analysed. Node **N**_15_ is a typical example of a node that may be unfairly categorised if the threshold values that determine the trustworthiness of a node are constant.

As observed in Figure 20, the computed $R_{\mathrm{Totalfp}}$ gradually increased as the simulation progressed which is likely due to more data packets being forwarded as observed by **N**_10_ and good second-hand reputations from node **N**_10_ neighbours about node **N**_15_. The high computed trust values of node **N**_15_ are a result of the accurate second-hand reputations it provided to node **N**_10_. This remained high and steady all through the simulations which are expected because node **N**_15_ was designated to always provide genuine second-hand reputations. As the simulation progressed the computed values of $R_{\mathrm{Totalfp}}$ gradually decreased while $R_{\mathrm{Totalmmp}}$ increased. The threshold, f, and s, which determine if a node is benevolent or selfish are set to be 0.75 and 0.25. This ensures that node **N**_15_ will be perceived as exhibiting a selfish behaviour between 390 → 400 s due to $R_{\mathrm{Totalfp}}$ dropping below 0.75 and $R_{\mathrm{Totaldp}}$ increasing above 0.25.

The evaluation process will be deemed fair if node **N**_15_ fails in all aspects such as

$$\{\begin{array}{c} R_{\mathrm{Totalfp}}<f\\ R_{\mathrm{Totaldp}}>s\\ R_{\mathrm{Totaldp}}>m\\ \omega_{ab}<\tau^{t} \end{array}$$

Assuming $\tau^{t}$ is given as 0.75. A situation where the computed trust values ($\omega_{ab}$) of node **N**_15_ as observed in Figure 20 is above 0.75, it may be unfair if node **N**_15_ is categorised as selfish and later penalised due to the computed $R_{\mathrm{Totalfp}}$ dropping slightly below f and the computed $R_{\mathrm{Totaldp}}$ increasing slightly above s. To maintain fairness in the trustworthy evaluation process of nodes from the computed total reputation and trust values as observed in Figure 20, the set of threshold values 〈f,s,m〉 should be within a given range. As long as the computed trust values $\omega_{ab}$ remains above the set threshold ${\;\tau }^{t}$, and the total reputation value measuring selfishness, $R_{\mathrm{Totaldp}}$, does not fall below the lower boundary of the given range (0.75, 0.5), a target node such as **N**_15_ should not be penalised and isolated from the network.

Evaluating the trustworthiness of a node using these conditions may not comprise the security of the network and will not undermine the idea of a trust and reputation system. Rather, the concept of candour is enshrined in the trustworthiness evaluation of nodes which is necessary due to the limitations associated with mobile nodes in MANETs. From the analyses of the simulation results, it was established that the trustworthiness of a node in the proposed model is evaluated using the novel candour two-dimensional trustworthiness evaluation technique. The first is the total reputation of a node which is measured from 3-tuples $\langle R_{\mathrm{Totalfp}},R_{\mathrm{Totaldp}},R_{\mathrm{Totalmmp}}\rangle$ represent benevolence, selfishness, and maliciousness respectively. The second view is the trust value $\omega_{ab}$ which measures the accuracy of second-hand reputations a node provides about other nodes. From the analysed computed total reputation values, it was established that for the candour concept to be enshrined in the trustworthiness evaluation of a node, it is necessary for the set threshold values that determines the categorisation of nodes to be set within a given range to accommodate for changing network situations to ensure that nodes are not unfairly penalised or isolated in the network. Various network scenarios were analysed from the computed reputation and trust values from the simulated behaviours of the network nodes. From the various scenarios analysed it was concluded that for fairness to be enshrined in the trustworthy evaluation process, the calculated total reputation and trust values of a target node must meet the following conditions:

$$\{\begin{array}{c} R_{\mathrm{Totalfp}}\;\;\;\in \;\left(0.5,\;0.75\right)\\ R_{\mathrm{Totaldp}}\;\;\;\in \;\left(0.25,\;0.5\right)\\ R_{\mathrm{Totaldp}}\;\;\;\leq \;\;\;\;\;\;\;\;\;\;\;\;\;\;\;\;0\\ {\;\omega }_{ab}\;\;\;\;\;\;\;\;\;\geq \;\;\;\;\;\;\;\;\;\;0.75 \end{array}$$

where the given values represent the optimal weights 〈f,s,m〉 for the thresholds that must be met before the trustworthiness of a node is established. The computed total reputation values $\langle R_{\mathrm{Totalfp}},R_{\mathrm{Totaldp}},R_{\mathrm{Totalmmp}}\rangle$ are evaluated such that:

(24) $$R_{\mathrm{Totalfp}}+R_{\mathrm{Totaldp}}+R_{\mathrm{Totalmmp}}=1$$

In all the scenarios in which the trustworthiness of a node in the network will be determined, the value of m is set as zero ($R_{\mathrm{Totalmmp}}$ < 0). This is to ensure that the proposed model does not tolerate or encourage the operations of malicious nodes. Selfish behaviours may be a direct result of a node’s physical properties (overloaded with forwarding requests, reduction in energy levels, and loss of battery power) which may be partially tolerated. On the other hand, malicious nodes may modify, inject or misroute packets. Their sole aim is to undermine security and integrity by attacking the network. This form of behaviour should not be tolerated in any form.

## 9. Conclusions

The proposed Dirichlet Reputation and Trust management system for Mobile Ad Hoc Networks system describes a novel candour two-dimensional trustworthiness evaluation technique based on what a node says about other nodes and what it does with regards to forwarding packets. The observed optimal weights at any given time were recommended to be used in evaluating the trustworthiness of a node which would ensure that nodes are not unfairly penalised especially if they can still contribute to the network passively (by providing genuine second-hand reputations about other nodes). The analysed simulation results established that the Dirichlet distribution is effective in modelling the behaviours of nodes which aided in understanding the behaviours of nodes without bias. In terms of using the observable optimal weights in evaluating the trustworthiness of a node in the network, it was established that to introduce and maintain candour in the trustworthy evaluation process of nodes from the computed total reputation and trust values, the set thresholds which determine the true nature of a node from the computed values should be within a given range. The developed Dirichlet Reputation and Trust management system model may involve additional cost computation. The analysis of our simulation results demonstrates that the proposed model can improve network security, reliability, and enshrine the candour concept in the trustworthiness evaluation of nodes which ensures that nodes are not unfairly penalised or isolated in the network. Therefore, we can conclude that to accomplish a reliable MANET using the proposed RTM model, the possible additional computational cost that the model will incur in the network serves as a compromise. Future research work will focus on incorporating priority queues in the proposed model. Priority queues for trustworthy nodes can be designed to ensure that packets from these nodes are sent out of the buffer before the packets from the other nodes. This will be solely aimed at rewarding the trustworthy nodes by enabling a better quality of service provisioning of their packets in the network.

## Figures and Tables

**Table 1:** Observed packet transmission activities over four defined monitoring intervals.

Monitoring Interval	α_1_	α_2_	α_3_	No. of Packets Observed	R_fp_	R_dp_	R_mmp_
1	45	5	0	50	0.900	0.100	0.000
2	27	3	0	30	0.900	0.100	0.000
3	35	5	0	40	0.875	0.125	0.000
4	32	8	0	40	0.800	0.200	0.000

**Table 2:** Second-hand reputations verification scenario.

Recommending Nodes	$\langle R_{fp2}\;,R_{dp2},R_{mmp2}\rangle$	Deviation (ϑ)	Test Result
C	〈0.880,0.120,0.000〉	0.113	Valid
D	〈0.850,0.150,0.000〉	0.071	Valid
E	〈0.900,0.100,0.000〉	0.141	Valid
F	〈0.550,0.450,0.000〉	0.354	Invalid

**Table 3:** Simulation environment and parameters.

Parameters	Values
**Topographical Area**	900 × 900 square metres
**Simulation time**	900 seconds
**Channel type**	Wireless Channel
**Radio-Propagation Mode**	TwoRayGround
**Antenna type**	OmniAntenna
**Routing Protocol**	AODV
**Interface queue type**	CMUPriQueue
**Maximum packet in Queue**	50 packets
**Network interface type**	Phy/WirelessPhy
**Link Layer Type**	LL
**MAC type**	802.11
**Number of Connections**	6
**Data Packet Size**	512 bytes
**Number of mobile nodes**	10, 20
**ϑ, γ, φ, σ**	0.30, 0.70, 0.30, 0.99

**Table 4:** Behaviours displayed by the various nodes.

Behaviours	Node-id
Good	**N**_0_, **N**_1_, **N**_2_, **N**_4_, **N**_6_, **N**_7_, **N**_9_
Periodically selfish	**N**_3_
Greyhole node	**N**_8_
Blackhole node	**N**_5_

**Table 5:** Behaviours displayed by the various nodes.

Behaviours	Accurate Second-Hand Reputations	Node-id
Good	Honest	**N**_0_, **N**_1_, **N**_2_, **N**_4_, **N**_6_, **N**_7_, **N**_9_
Periodically selfish	Dishonest	**N**_3_
Greyhole node	Dishonest	**N**_8_
Blackhole node	Dishonest	**N**_5_

**Table 6:** Behaviours displayed by the various nodes.

Packet Forwarding Behaviour	Accuracy of Second-Hand Reputations	Node-id
Good	Honest	**N**_0_, **N**_1_, **N**_2_, **N**_3_, **N**_4_
Good	Honest	**N**_7_, **N**_8_, **N**_10_, **N**_13_, **N**_13_, **N**_17_
Good	Dishonest	**N**_6_, **N**_9_
Periodically Selfish	Dishonest	**N**_12_, **N**_14_, **N**_16_
Low Energy-Constrained Selfish	Honest	**N**_5_, **N**_11_, **N**_15_, **N**_19_

## Data Availability

The data presented in this study are available on request from the corresponding author.
